# Arbuscular Mycorrhizal Symbiosis Modulates Antioxidant Response and Ion Distribution in Salt-Stressed *Elaeagnus angustifolia* Seedlings

**DOI:** 10.3389/fmicb.2018.00652

**Published:** 2018-04-05

**Authors:** Wei Chang, Xin Sui, Xiao-Xu Fan, Ting-Ting Jia, Fu-Qiang Song

**Affiliations:** ^1^College of Forestry, Northeast Forestry University, Harbin, China; ^2^College of Life Sciences, Heilongjiang University, Harbin, China

**Keywords:** arbuscular mycorrhizal symbiosis, *Elaeagnus angustifolia* L., antioxidant enzyme, reactive oxygen species (ROS), mineral nutrient, dry weight, atomic absorbance spectrophotometer

## Abstract

*Elaeagnus angustifolia* L. is a drought-resistant species. Arbuscular mycorrhizal symbiosis is considered to be a bio-ameliorator of saline soils that can improve salinity tolerance in plants. The present study investigated the effects of inoculation with the arbuscular mycorrhizal fungus *Rhizophagus irregularis* on the biomass, antioxidant enzyme activities, and root, stem, and leaf ion accumulation of *E. angustifolia* seedlings grown during salt stress conditions. Salt-stressed mycorrhizal seedlings produced greater root, stem, and leaf biomass than the uninoculated stressed seedlings. In addition, the seedlings colonized by *R. irregularis* showed notably higher activities of superoxide dismutase (SOD), catalase (CAT), and ascorbate peroxidase (APX) in the leaves of the mycorrhizal seedlings in response to salinity compared to those of the non-mycorrhizal seedlings. Mycorrhizal seedlings not only significantly increased their ability to acquire K^+^, Ca^2+^, and Mg^2+^, but also maintained higher K^+^:Na^+^ ratios in the leaves and lower Ca^2+^:Mg^2+^ ratios than non-mycorrhizal seedlings during salt stress. These results suggest that the salt tolerance of *E. angustifolia* seedlings could be enhanced by *R. irregularis.* The arbuscular mycorrhizal symbiosis could be a promising method to restore and utilize salt-alkaline land in northern China.

## Introduction

The salinization of soils is a major ecological and agronomic problem, affecting approximately one billion hectares of arid and semiarid areas in the world (Dagar and Minhas, 2016). Salinity is considered to be one of the most important abiotic stresses that affects the establishment, growth, and development of plants, causing important biomass production losses in most of the arid and semiarid regions of the world (Evelin et al., 2009). During salt stress conditions, plant physiology is often affected in the following manner: (a) excessive amounts of ions, such as Na^+^, cause the destruction of enzyme structures and cell organelles, thereby disrupting photosynthesis, respiration, and protein synthesis in plants (Feng et al., 2002; Ruiz-Lozano et al., 2012); (b) accumulation of salts in the soil induces physiological drought and nutrient imbalance in plants (Feng et al., 2002; Ruiz-Lozano et al., 2012); and (c) salinity stress causes the plants to produce reactive oxygen species (ROS) (Mittler, 2002).

*Elaeagnus angustifolia* L. (Russian olive) is able to grow in a wide range of climates and soil conditions, particularly on disturbed sites, and is one of the native dominant species in the arid region of northwest China (Zhao et al., 2006). As an important economic tree species, *E. angustifolia* is used for its fruit, fuel wood, gum, leaf fodder, nectar and honey, medicinal properties, and is also planted for its beauty (Khamzina et al., 2009; Hamidpour et al., 2017).

Abuscular mycorrhizal (AM) fungi are associated with most terrestrial plant species including halophytes, hydrophytes, and xerophytes (Evelin et al., 2009). Many studies have demonstrated that AM symbiosis is one of the strategies that plants use to grow during a variety of abiotic stress conditions such as low temperature stress, droughts, and salt stress (Lopez-Raez, 2016). AMF can promote the host plant’s resistance to salinity by using various mechanisms such as the regulation of plant physiology and development (Borde et al., 2011; Aroca et al., 2013; Bharti et al., 2013; Estrada et al., 2013a,b), the expression of stress-related genes and proteins (Aroca et al., 2009; Evelin et al., 2009; Ruiz-Lozano et al., 2012), enhanced nutrient acquisition and water uptake (Evelin et al., 2009; Chandrasekaran et al., 2014), the defense of roots against soil-borne pathogens (Azcón-Aguilar et al., 2002) and the production of a large amount of external mycelia that increase the soil exploration capacity (Evelin et al., 2009). Therefore, many researchers have suggested that AMF might be effective candidates to use in the bio-amelioration of saline soils (Giri et al., 2007).

The exposure of plants to environmental and biotic stress enhances the accumulation of toxic ROS such as the superoxide radical (O_2_^-^), hydrogen peroxide (H_2_O_2_), hydroxyl radical (OH^-^) and singlet oxygen (^1^O_2_) (Mittler, 2002; Abd Allah et al., 2015). ROS can severely disrupt the normal metabolism and cellular functions of plants through its oxidative damage to lipids, nucleic acids, oxidizing proteins, and photosynthetic pigments (Abd Allah et al., 2015). The induction of ROS-scavenging enzymes, such as superoxide dismutase (SOD), catalase (CAT), peroxidase (POD), ascorbate peroxidase (APX), and glutathione reductase (GR) are the most common mechanisms used to scavenge toxic ROS during salt stress (Evelin and Kapoor, 2014; Demidchik, 2015). Several studies have demonstrated that AM symbiosis can serve to protect the host plants against oxidative damage during salt stress (Ghorbanli et al., 2004; He et al., 2007; Garg and Manchanda, 2009; Hajiboland et al., 2009; Wu et al., 2010; Abdel Latef and Chaoxing, 2011; Borde et al., 2011; Estrada et al., 2013a). Higher antioxidant enzyme activity in mycorrhizal plants helps to rapidly and efficiently remove excess ROS. However, no change or decrease in the activities of SOD, CAT, POD, and APX have been reported in mycorrhizal soybean, bajra, and tomato during salinity stress.

As the first barrier for ion selection, AM fungi can selectively absorb elements, such as K^+^, Ca^2+^, and Mg^2+^, while avoiding Na^+^ uptake (Cantrell and Linderman, 2001; Hammer et al., 2011; Estrada et al., 2013b). Several studies have shown that the AM symbiosis can prevent Na^+^ transfer to shoot tissues under saline conditions while enhancing K^+^ uptake (Cantrell and Linderman, 2001; Giri et al., 2007; Sharifi et al., 2007; Talaat and Shawky, 2011; Estrada et al., 2013b). Thus, mycorrhizal plants often have a higher K^+^:Na^+^ ratio during salinity (Rabie, 2005; Sannazzaro et al., 2006; Giri et al., 2007) and a lower shoot Na^+^ concentration than non-mycorrhizal plants (Al-Karaki, 2000). Ca^2+^ is important to maintain the integrity and structures of membranes and cell walls (Cramer, 2002). Ca^2+^ is also used as a secondary messenger in many signal transduction pathways within the cells (Cramer, 2002). The Ca^2+^:Na^+^ ratio can affect growth, photosynthesis, plant nutrition, and water and ion transport in plants (Cramer, 2002). Therefore, the Ca^2+^:Na^+^ ratio is also one of the key determinants of plant salt tolerance. In addition, it has been proposed that additional Ca^2+^ can alleviate salinity-induced growth reduction (Lazof and Bernstein, 1999).

Thus far, no studies have focused on the mechanisms of mycorrhizal *E. angustifolia* plants to alleviate salt stress. In this study, we monitored the ion distributions of Na^+^, K^+^, Ca^2+^, and Mg^2+^ and the activities of SOD, POD, APX, and CAT in the leaves of AM and non-AM *E. angustifolia* in 300 mmol/L NaCl and normal conditions. The objective of this study was to evaluate the effects of AMF on the antioxidant response and ion distribution of *E. angustifolia* plants in a saline environment to better understand salt tolerance mechanisms in AM plants.

## Materials and Methods

### Experimental Design

The experiment was laid out in a randomized complete block design with three factors: (1) non-mycorrhizal control; (2) inoculation with the AM fungus *Rhizophagus irregularis* (previously known as *Glomus intraradices*), and (3) two salinity levels of 0 and 300 mM NaCl. Each of the four treatments had eight replicates. The positions of the pots were changed every week to eliminate environmental error.

### Plant Materials and Soil

*E. angustifolia* seeds were surface-sterilized for 10 min in 0.2% KMnO_4_, rinsed four times with sterile distilled water, and grown in plastic pots (5 L) containing substrate (soil:vermiculite 3:1, V/V), previously sterilized in an autoclave for 1 h at 121°C three times on alternate days. The soil was collected from the Forest Botanical Garden of Heilongjiang Province (China, 45° 42′ 40.09″ N 126° 38′ 22.23″ W), sieved (5 mm), and diluted with vermiculite (3:1, soil:vermiculite, V/V). The original substrate had a pH of 7.2 (measured in water 1:5 [w/v]); 1.2% organic matter, nutrient concentrations (mg/kg): effective nitrogen, 123.4; available phosphorus, 12.6; available potassium, 76.5. The electrical conductivity of the original soil was 0.5 dS/m.

### Inoculation Treatments

The mycorrhizal inocula consisted of soil, spores, mycelia, and infected root fragments that contained approximately 25–30 AM propagules/g, and were obtained from an open pot culture (*Amorpha fruticosa* L.) of *R. irregularis* (synonym of *G. intraradices* DAOM 197198) (Kruger et al., 2012), previously isolated from the Zhao YueShan National Wetland Park (Heilongjiang Province, China, 46° 5′ 37.98″ N 125° 57′ 28.43″ W), which is an area with severe salinity problems.

The soil was inoculated with *R. irregularis* at the time of sowing. The inoculated dosage of mycorrhizal inoculum per pot was 10 g, which was planted 2 cm below the surface of the soil. Non-inoculated control pots received the same amount of autoclaved mycorrhizal inocula in order to provide the same microbial population free of AM propagules.

### Saline Stress

Water was supplied daily during the entire period of plant growth to avoid any drought effects. The plants were established for approximately 100 days prior to salinization to allow adequate plant growth and symbiotic establishment.

Two concentrations (0 and 300 mmol/L NaCl) of saline solution were added to the soil substrate by adding pre-determined amounts of NaCl from a 2 mol/L stock saline solution based on the amount of substrate in the pots. The concentration of NaCl in the soil was increased gradually on alternate days to avoid osmotic shock. It took 6 days to reach the desired levels of 300 mmol/L NaCl. Seedlings were maintained under these conditions for 30 additional days. The experiment was carried out under outdoor natural conditions.

### Measurement of AM Colonization and Dry Weight

The harvested seedlings were washed with distilled water three times. For each plant, the roots, stems and leaves were dried for 2 days to a constant weight at 70°C. The dry weight of the roots, stems, and leaves were recorded.

The fine roots were cut into 1-cm segments, cleared in 10% KOH, and stained with 0.05% trypan blue. Thirty fragments were examined for AM colonization under a digital computerized microscope (Model DP-73, Olympus). All AM fungal structures including hyphae, arbuscules, and vesicles found in the roots were recorded. The mycorrhizal percentage was determined by the method of McGonigle et al. (1990).

### Measurement of Antioxidant Enzyme Activities

Extracts to determine the activities of SOD, POD, CAT, and APX used 0.5 g of fresh leaves frozen in liquid nitrogen that were then homogenized in 4 ml of solution containing 50 mmol/L Tris-HCl buffer (pH 7.5) and 0.2 mmol/L ascorbic acid. The homogenized samples were centrifuged at 4°C and 13,000 × *g* for 20 min, and then the supernatants were used to analyze SOD, POD, CAT, and APX activity and measure the amount of total protein using commercial assay kits (Nanjing Jiancheng Bioengineering Institute, Nanjing, CHN). The detailed procedures were performed as per the manufacturer’s instruction. All enzymes and total protein above were detected using a UV/VIS Spectrophotometer.

Total protein levels were measured by the BCA method using a total protein assay kit (Nanjing Jiancheng Bioengineering Institute, Nanjing, CHN). The principle of this method was that proteins can reduce Cu^2+^ to Cu^+^ under alkaline conditions. The amount of reduction was proportional to the amount of protein present. BCA formed a purple-blue complex with Cu^+^ in alkaline environments, thus providing a basis to monitor the reduction of alkaline Cu^2+^ by the proteins. The absorbance of each reaction mixture was measured at 562 nm.

The activity of SOD (EC 1.15.1.1) was determined by the xanthine oxidase method using the T-SOD activity assay kit (Nanjing Jiancheng Bioengineering Institute, Nanjing, CHN). Briefly, SOD activity was tested by measuring its inhibition on the process of xanthine oxidase catalyzing xanthine to generate superoxide anion free radicals. The superoxide radicals could then oxidize hydroxylamine to generate nitrite, which reacts with a developer to give a purple color. The absorbance of each reaction mixture was measured at 550 nm. One unit of SOD activity was defined as the quantity of SOD required to produce 50% inhibition of reduction of nitrite in a 1 mL reaction solution.

The activity of CAT (EC 1.11.1.6) was detected using the CAT assay kit (Nanjing Jiancheng Bioengineering Institute, Nanjing, CHN). Briefly, the assay principle was based on the reaction of catalase to decompose H_2_O_2_, which absorbs maximally at 240 nm. CAT activity was calculated by the decrease in absorbance at 240 nm due to the decomposition of H_2_O_2_. One unit of CAT activity was defined as the amount of enzyme that caused the decomposition of 1 mmol H_2_O_2_ per second at 25°C in 1 g tissue protein.

The activity of POD (EC 1.11.1.7) was measured following the change of absorption at 420 nm using the POD assay kit (Nanjing Jiancheng Bioengineering Institute, Nanjing, CHN). In the assay, peroxidase reacted with pyrogallol to produce purpurogallin, which can be measured at 420 nm. One unit of POD activity was defined as the amount of enzyme that catalyzed 1 μg of substrate by 1 mg of tissue protein per minute at 37°C in the reaction system.

The activity of APX (EC 1.11.1.11) was measured using the APX assay kit (Nanjing Jiancheng Bioengineering Institute, Nanjing, CHN). Briefly, APX utilized ascorbic acid (AsA) with a maximum absorption of 290 nm as its specific electron donor to reduce H_2_O_2_ to water with the concomitant generation of monodehydroascorbate (MDAsA). Ascorbate peroxidase activity was thus determined by the decrease in absorbance at 290 nm due to the oxidation of AsA. One unit of APX activity was defined as the amount of enzyme that catalyzed 1 μmol ASA using 1 mg of tissue protein per minute at 37°C in the reaction system.

Superoxide dismutase, APX, POD, and CAT activities were calculated according to the manufacturer’s instructions. The activities of SOD, APX, and POD are expressed as U/mg protein. The activity of CAT is presented as U/g protein, where U represents the units of enzymatic activity.

### Determination of Mineral Nutrients

Leaf, stem, and root samples were blotted dry on filter paper and dried at 70°C for 2 days to a constant weight. Na^+^, K^+^, Ca^2+^, and Mg^2+^ ions were extracted from 0.1 g of ground leaf, stem, and root dry matter to perform the analyses. Five milliliters of acid mixture consisting of HNO_3_ and HClO_4_ at a ratio of 4:1 was added to the samples and incubated at 60°C overnight. The samples were then mixed with 4 mL HNO_3_^+^ and 1mL H_2_O_2_, heated to 220°C for 20 min, and cooled at room temperature for at least 4 h. After that, the extraction samples were diluted with deionized water and injected into an Atomic Absorbance Spectrophotometer (AA-6650, SHIMADZU [CHINA] CO., LTD., JP) for the analysis. Three different plants for each treatment were extracted. The blank was run without plant samples.

### Statistical Analysis

#### Principal Component Analysis (PCA) and Heatmaps

The data were first standardized and then used for variance decomposition to reflect the difference between multiple sets of data on a two-dimensional coordinate map, and the coordinate axes used two eigenvalues that can reflect the maximum variance value. PCA was performed on the CANOCO 4.5 software. Pearson correlation distance-matrix was analyzed by SPSS 13.0 and then the heatmap was generated by R software.

The data were analyzed using the statistical software SPSS 13.0 (SPSS Inc., Chicago, IL, United States) and Graph Pad Prism 6. The data were statistically analyzed by one-way and two-way analysis of variance (ANOVA) with NaCl treatment, M inoculation, and interactions among them as sources of variation. Multiple comparisons of the means were performed by the Uncorrected Fisher’s LSD test (*p* < 0.05).

## Results

### Colonization of AMF in the Plant Roots

**FIGURE 1:**
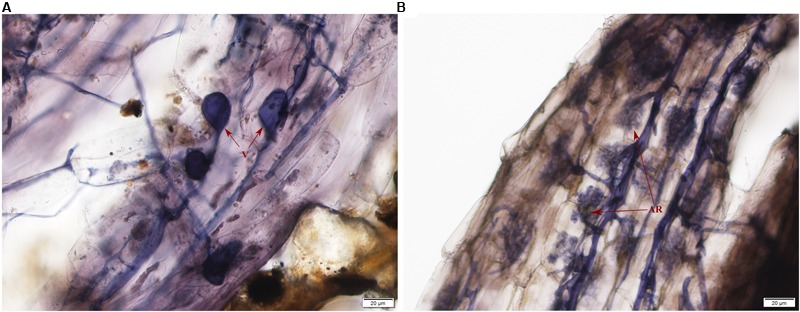
Photomicrographs of structural colonization of arbuscular mycorrhizal fungi in the roots of *Elaeagnus angustifolia*. **(A)** Vesicles (V); **(B)** Arbuscule (AR).

Typical AMF morphological structures were detected in inoculated *E. angustifolia* fine roots including vesicles (**Figure 1A**) and arbuscules (**Figure 1B**). The maximum AM colonization percentage of the root reached 96% at approximately 100 days after inoculation. The 96% of AM colonization demonstrated that *E. angustifolia* and *R. irregularisi* established a vigorous symbiosis before the salt stress treatment. No colonization was found in the non-inoculated seedlings.

### Dry Weights of Roots, Stems, and Leaves in the Mycorrhizal and Non-mycorrhizal Seedlings During Salt Stress

Salt stress decreased the root, stem, and leaf dry weights in the mycorrhizal seedlings, but the mycorrhizal seedlings grew better than the non-mycorrhizal seedlings during salt stress (**Table 1**). During the 300 mmol/L NaCl treatment, the root, stem, and leaf dry weights of the mycorrhizal seedlings increased by 35.7, 13.6, and 53.3%, respectively, compared with those of the non- mycorrhizal seedlings. AMF inoculation significantly enhanced the survival of *E. angustifolia* seedlings in the presence of 300 mmol/L NaCl.

**Table 1:** The effects of salt and arbuscular mycorrhizal fungi (AMF) on the dry weight of roots, stems, and leaves of *Elaeagnus angustifolia* seedlings.

Treatments		Root dry weight (g plant^-1^)	Stem dry weight (g plant^-1^)	Leaf dry weight (g plant^-1^)
0 mmol/L	NM	0.34 ± 0.10^bc^	0.49 ± 0.08^bc^	0.26 ± 0.02^ab^
	M	0.62 ± 0.13^a^	0.78 ± 0.10^a^	0.31 ± 0.04^a^
300 mmol/L	NM	0.28 ± 0.04^c^	0.44 ± 0.01^c^	0.15 ± 0.04^c^
	M	0.38 ± 0.03^b^	0.50 ± 0.05^b^	0.23 ± 0.04^b^
**Significance**				
Salt		^∗∗^	^∗∗^	^∗∗^
Mycorrhizal		^∗∗^	^∗∗^	^∗∗^
Salt × Mycorrhizal		^∗^	^∗∗^	NS

*M, mycorrhizal; NM, non-mycorrhizal; 0 mmol/L, without salt stress; 300 mmol/L, during salt stress.*

*Data are means ± SD of six replicates. The same letter within each column shows no significant differences among treatments (p < 0.05). Levels of significance: ^∗^p < 0.05, ^∗∗^p < 0.01. NS, not significant.*

### Antioxidant Enzyme Activities in Leaves in the Mycorrhizal and Non-mycorrhizal Seedlings During Salt Stress

**FIGURE 2:**
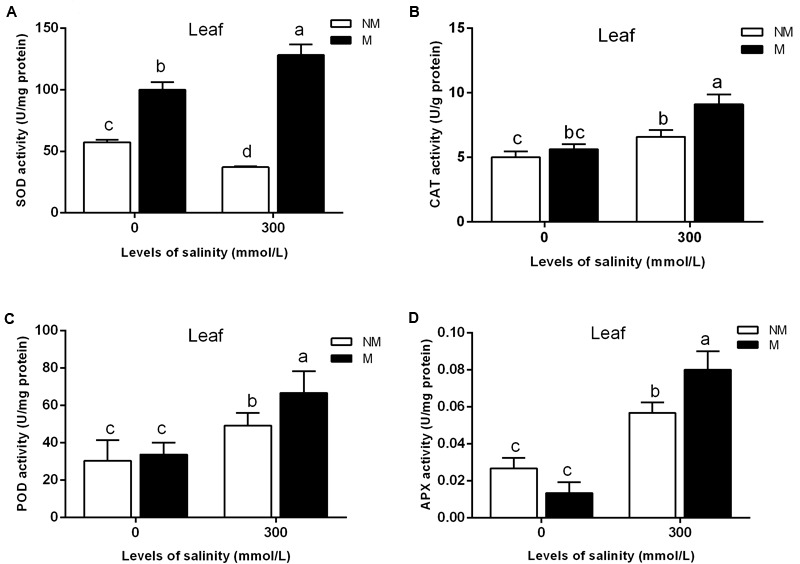
Effects of arbuscular mycorrhizal fungi inoculation on the superoxide dismutase (SOD) **(A)**, catalase (CAT) **(B)**, peroxidase (POD) **(C)**, and ascorbate peroxidase (APX) **(D)** activities in the leaves during different salt conditions. M, mycorrhizal; NM, non-mycorrhizal; 0 mmol/L, without salt stress; 300 mmol/L, during salt stress. Columns represent the means for three plants (*n* = 3). Error bars show the standard error. Columns with different letters indicate significant differences between treatments at *P* < 0.05.

As shown in **Figure 2A**, AMF inoculation significantly promoted leaf SOD activity in the treatments lacking salt. The SOD activity in the leaves of mycorrhizal seedlings increased significantly during salt stress, but that in the non-mycorrhizal seedlings declined during salt stress (**Figure 2A**). Mycorrhizal seedlings had a higher leaf SOD activity than that of the non-mycorrhizal seedlings. Compared with that of non-mycorrhizal seedlings, the leaf SOD activity of mycorrhizal seedlings increased by 245% during salt stress. A two-way ANOVA also revealed significant effects of AMF and M × S on the SOD activity (**Table 2**).

**Table 2:** Two-way analysis of variance (ANOVA).

Parameter measured	Significance of sources of variation		
	Salt treatment (S)	Mycorrhizal (M)	M × S
APX	^∗∗∗^	NS	^∗∗^
CAT	^∗∗∗^	^∗∗∗^	^∗^
POD	^∗∗∗^	NS	NS
SOD	NS	^∗∗∗^	^∗∗∗^
Root K^+^ concentration	^∗∗∗^	^∗∗∗^	^∗∗∗^
Stem K^+^ concentration	NS	^∗∗^	^∗∗^
Leaf K^+^ concentration	^∗∗∗^	^∗∗∗^	^∗∗∗^
Root Na^+^ concentration	^∗∗∗^	^∗∗∗^	^∗∗∗^
Stem Na^+^ concentration	^∗∗∗^	^∗∗^	^∗∗^
Leaf Na^+^ concentration	^∗∗∗^	^∗^	^∗∗∗^
Root Ca^2+^ concentration	^∗∗∗^	^∗∗∗^	^∗∗∗^
Stem Ca^2+^ concentration	^∗∗∗^	^∗∗^	^∗∗∗^
Leaf Ca^2+^ concentration	^∗∗∗^	^∗∗∗^	^∗∗∗^
Root Mg^2+^ concentration	^∗∗∗^	^∗∗^	^∗∗∗^
Stem Mg^2+^ Concentration	^∗^	^∗∗∗^	^∗∗∗^
Leaf Mg^2+^ concentration	^∗∗∗^	^∗∗∗^	NS
Root K^+^/Na^+^ ratio	^∗∗∗^	NS	NS
Stem K^+^/Na^+^ ratio	^∗∗∗^	NS	NS
Leaf K^+^/Na^+^ ratio	^∗∗∗^	NS	^∗∗^
Root Ca^2+^/Na^+^ ratio	^∗∗∗^	NS	NS
Stem Ca^2+^/Na^+^ ratio	^∗∗∗^	NS	NS
Leaf Ca^2+^/Na^+^ ratio	^∗∗∗^	NS	NS
Root Ca^2+^/Mg^2+^ ratio	^∗∗∗^	^∗∗^	^∗∗∗^
Stem Ca^2+^/Mg^2+^ ratio	^∗∗∗^	^∗∗∗^	^∗∗^
Leaf Ca^2+^/Mg^2+^ ratio	^∗^	^∗∗∗^	^∗∗∗^

*The sources of variation are mycorrhizal inoculation (M), salt treatment (S), and their interaction (M × S). ^∗^P < 0.05, ^∗∗^P < 0.01, ^∗∗∗^P < 0.001; NS, not significant.*

Salinity stress caused a significant increase in the leaf CAT activity of mycorrhizal and non-mycorrhizal seedlings. The mycorrhizal and non-mycorrhizal seedlings had a similar leaf CAT activity during no-salt stress treatments, while mycorrhizal seedlings had a higher leaf CAT activity than non-mycorrhizal seedlings during salt stress treatments (**Figure 2B**). The leaf CAT activity increased by 38.0% in the mycorrhizal seedlings compared with the non-mycorrhizal seedlings during salt stress conditions. A two-way ANOVA revealed a significant influence of NaCl, AMF, and their interactions on the CAT activity (**Table 2**).

The APX activity of the mycorrhizal and non-mycorrhizal seedling leaves was markedly increased by salt stress (**Figure 2D**). In the leaves, AM colonization only increased the APX activity in mycorrhizal seedlings during salinity stress, and no significant differences between the mycorrhizal and non-mycorrhizal treatments were found in the APX activity of leaves when the plants were not subjected to salt stress (**Figure 2D**). The APX activity of the leaves was 40.3% higher in the mycorrhizal than in the non-mycorrhizal seedlings during salt stress conditions. A two-way ANOVA revealed a significant influence of NaCl and M × S on the APX activity (**Table 2**).

The POD activity of leaves was significantly enhanced by salt stress (**Figure 2C**). However, there was no significant effect of AMF and M × S on the POD activity (**Table 2**).

The mycorrhization of the plants led to increased levels of leaf antioxidant defense systems during stress conditions.

### Na^+^, K^+^, Ca^2+^, and Mg^2+^ Contents in Roots, Stems, and Leaves in the Mycorrhizal and Non-mycorrhizal Seedlings During Salt Stress

**FIGURE 3:**
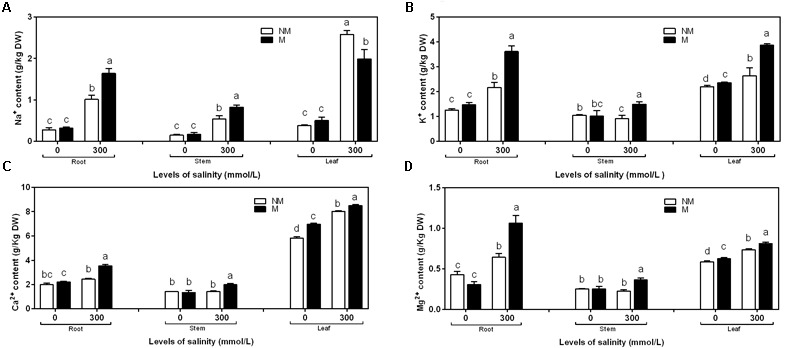
Na^+^ **(A)**, K^+^ **(B)**, Ca^2+^ **(C)**, and Mg^2+^ **(D)** concentrations in the root, stem, and leaf tissues of mycorrhizal and non-mycorrhizal seedlings grown in different salt conditions. M, mycorrhizal; NM, non-mycorrhizal; 0 mmol/L, without salt stress; 300 mmol/L, during salt stress. Columns represent the means for three plants (*n* = 3). Error bars show the standard error. Columns with different letters indicate significant differences between the treatments at *P* < 0.05.

In the case of roots, stems, and leaves, the concentration of Na^+^ was similar in the mycorrhizal and non-mycorrhizal seedlings that were not treated with salt. Mycorrhizal seedlings had less Na^+^ than non-mycorrhizal seedlings at 300 mmol/L salt levels in the leaves, but it had more Na^+^ than non- mycorrhizal seedlings at 300 mmol/L salt levels in the roots and stems (**Figure 3A**). The two independent factors, NaCl and AMF, and the interactions between and among them displayed significant effects on the concentration of Na^+^ (**Table 2**).

NaCl stress significantly increased the K^+^ content compared to mycorrhizal and non-mycorrhizal seedlings during non-stressed conditions in the roots, stems, and leaves. During non-saline conditions, the mycorrhizal seedlings accumulated slightly more K^+^ in the leaves than the non-mycorrhizal seedlings, while the K^+^ accumulation in the roots and stems was similar in the mycorrhizal and non-mycorrhizal seedlings (**Figure 3B**). A two-way ANOVA revealed a significant influence of NaCl, AMF, and M × S on the K^+^ content (**Table 2**).

During salinity treatments, mycorrhizal seedlings had significantly higher Ca^2+^ and Mg^2+^ concentrations in the roots, stems, and leaves compared to the non-mycorrhizal seedlings. In the roots and stems, the concentrations of Ca^2+^ and Mg^2+^ were similar between the mycorrhizal and non-mycorrhizal seedlings that were not subjected to salt treatment. AM seedlings had significantly higher Ca^2+^ and Mg^2+^ concentrations in leaves compared to the non-mycorrhizal seedlings that lacked salt stress (**Figures 3C,D**). A two-way ANOVA revealed that the Ca^2+^ and Mg^2+^ concentrations were significantly influenced by NaCl, AMF, and their interactions (**Table 2**).

### K^+^:Na^+^, Ca^2+^:Na^+^, and Ca^2+^:Mg^2+^ Ratios in the Roots, Stems, and Leaves in the Mycorrhizal and Non-mycorrhizal Seedlings During Salt Stress

**FIGURE 4:**
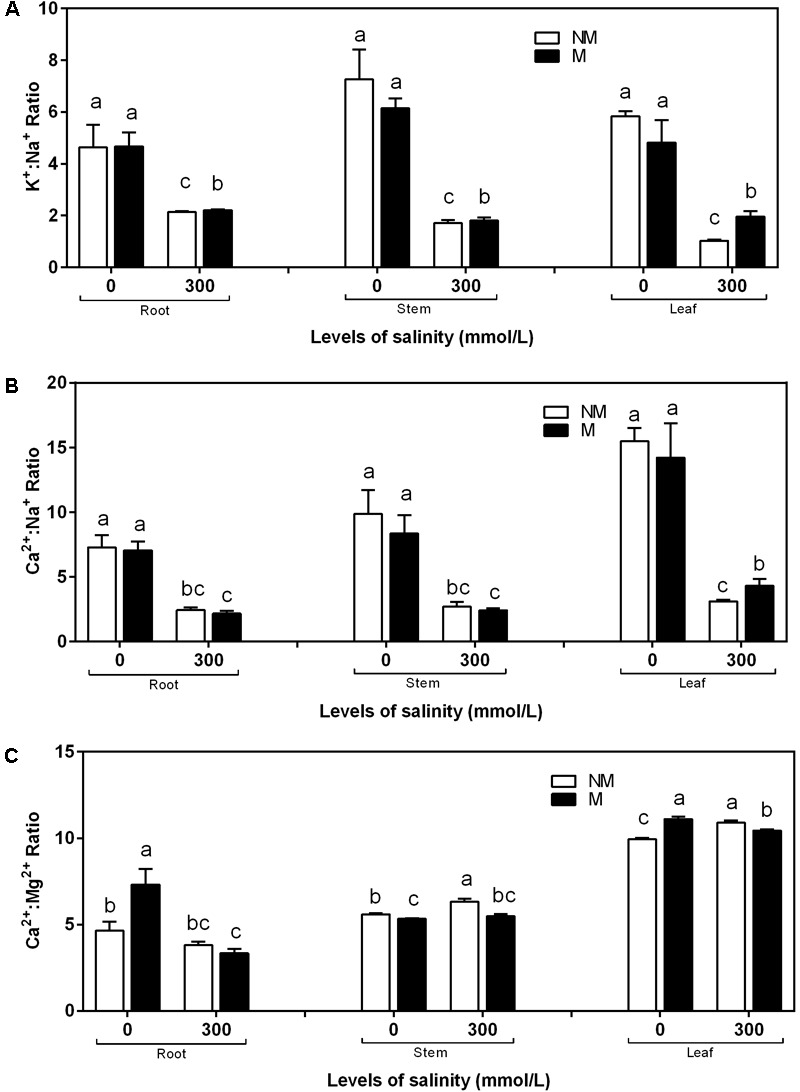
K^+^:Na^+^**(A)**, Ca^2+^:Na^+^ **(B)**, and Ca^2+^:Mg^2+^ **(C)** ratios of the root, stem, and leaf tissues in mycorrhizal and non-mycorrhizal seedlings during different salt conditions. M, mycorrhizal; NM, non-mycorrhizal; 0 mmol/L, without salt stress; 300 mmol/L, during salt stress. Columns represent the means for three plants (*n* = 3). Error bars show the standard error. Columns with different letters indicate significant differences between treatments at *P* < 0.05.

The K^+^:Na^+^ and Ca^2+^:Na^+^ ratios in the roots, stems, and leaves was affected considerably by salinity stress (**Figures 4A,B**). During non-saline conditions, the K^+^:Na^+^ and Ca^2+^:Na^+^ ratios in the roots, stems, and leaves showed no significant differences between the mycorrhizal and non-mycorrhizal seedlings. The mycorrhizal seedlings increased the K^+^:Na^+^ and Ca^2+^:Na^+^ ratios in the leaves during salt stress compared to the non-mycorrhizal seedlings. A two-way ANOVA revealed that the K^+^:Na^+^ ratio in the leaves was significantly influenced by NaCl and M × S (**Table 2**). In the roots and stems of the mycorrhizal seedlings, the K^+^:Na^+^ ratios at 300 mmol/L NaCl were higher than those in the non-mycorrhizal seedlings, and the Ca^2+^:Na^+^ ratios of the mycorrhizal seedlings were similar to those of the non-mycorrhizal seedlings (**Figures 4A,B**). However, there were no significant effects of AMF and M × S on the K^+^:Na^+^ and Ca^2+^:Na^+^ ratios in the roots and stems (**Table 2**). The Ca^2+^:Mg^2+^ ratio in the stems and leaves of the mycorrhizal seedlings was lower than that of the non-mycorrhizal seedlings during salinity stress (**Figure 4C**). A two-way ANOVA revealed that the Ca^2+^:Mg^2+^ concentrations were significantly influenced by NaCl, AMF, and their interactions (**Table 2**).

### Na^+^(Stem+Leaf):Na^+^(Root) Ratios in the Mycorrhizal and Non-mycorrhizal Seedlings During Salt Stress

**FIGURE 5:**
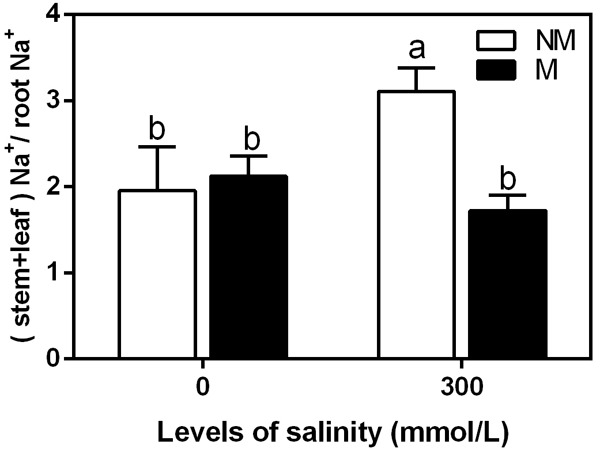
Na^+^(stem+leaf):Na^+^(root) ratio in mycorrhizal and non-mycorrhizal seedlings during different salt conditions. M, mycorrhizal; NM, non-mycorrhizal; 0 mmol/L, without salt stress; 300 mmol/L, during salt stress. Columns represent the means for three plants (*n* = 3). Error bars show the standard error. Columns with different letters indicate significant differences between the treatments at *P* < 0.05 by the Duncan’s test.

The Na^+^(stem+leaf) to Na^+^ (root) ratio is indicative of Na^+^ translocation to the shoots. This ratio tended to increase during salinity in the non-mycorrhizal seedlings (**Figure 5**). However, it showed no significant differences in the mycorrhizal seedlings. Under non-saline conditions, there were no significant differences in the ratio between the mycorrhizal and non-mycorrhizal seedlings. During the 300 mmol/L NaCl treatment, the ratio was 44% lower in the mycorrhizal seedlings than in the non-mycorrhizal seedlings.

### Correlation of Antioxidant Enzyme Activities and Ion Distribution in the Mycorrhizal and Non-mycorrhizal Seedlings During Salt Treatments

**FIGURE 6:**
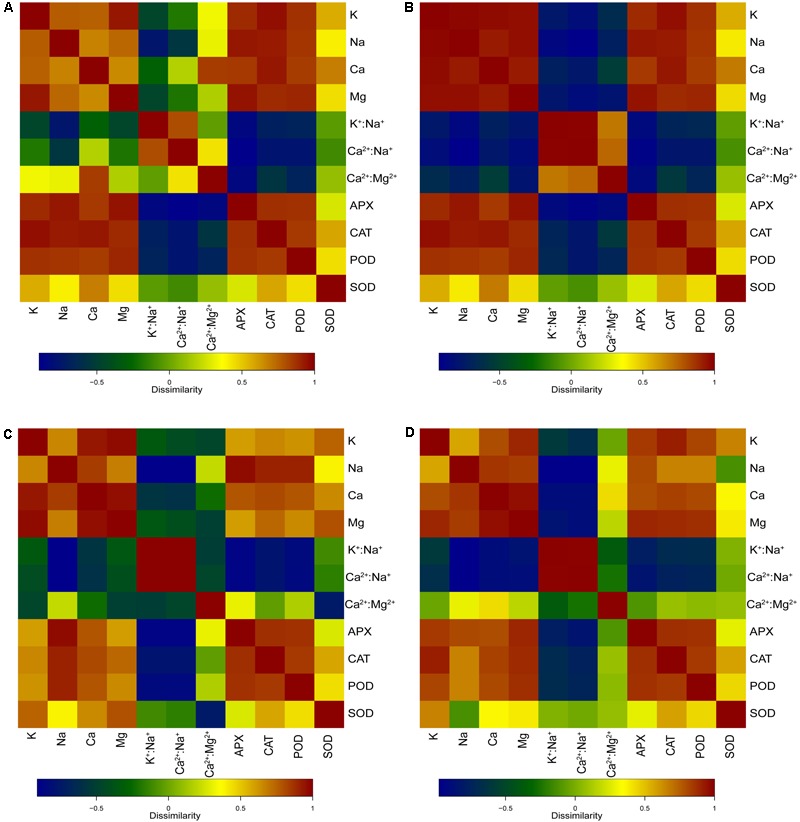
Correlation heatmaps of antioxidant enzyme activities and ion contents in different salt concentration treatments. **(A)** Correlation heatmap of antioxidant enzyme activities [superoxide dismutase (SOD), peroxidase (POD), catalase (CAT), and ascorbate peroxidase (APX)] in leaves and ion contents (Na^+^, K^+^, Ca^2+^, and Mg^2+^), ion ratios (K^+^:Na^+^, Ca^2+^:Na^+^, and Ca^2+^:Mg^2+^) in the whole seedling plant. **(B)** Correlation heatmap of antioxidant enzyme activities (SOD, CAT, POD, and APX) in leaves, and ion contents (Na^+^, K^+^, Ca^2+^, and Mg^2+^) and ion ratios (K^+^:Na^+^, Ca^2+^:Na^+^, and Ca^2+^:Mg^2+^) in roots. **(C)** Correlation heatmap of antioxidant enzyme activities (SOD, CAT, POD, and APX) in leaves and ion contents (Na^+^, K^+^, Ca^2+^, and Mg^2+^), ion ratios (K^+^:Na^+^, Ca^2+^:Na^+^, and Ca^2+^:Mg^2+^) in stems. **(D)** Correlation heatmap of antioxidant enzyme activities (SOD, CAT, POD, and APX) in leaves and ion contents (Na^+^, K^+^, Ca^2+^, and Mg^2+^), ion ratios (K^+^:Na^+^, Ca^2+^:Na^+^, and Ca^2+^:Mg^2+^) in leaves.

As shown in **Figure 6A**, the POD, APX, and CAT activities of the leaves were significantly positively related with Na^+^ content (*p* < 0.01), K^+^ content (*p* < 0.01), Ca^2+^ content (*p* < 0.01), and Mg^2+^content (*p* < 0.01) in the whole seedling during different salt concentration treatments. The SOD activity was significantly positively related with Ca^2+^ content (*p* < 0.05). The POD and APX activities of the leaves showed significant negative relationship with K^+^:Na^+^ (*p* < 0.05 and *p* < 0.01), Ca^2+^:Na^+^ (*p* < 0.01 and *p* < 0.01), and Ca^2+^:Mg^2+^ (*p* < 0.05 and *p* < 0.01) in the whole seedling during different salt concentration treatments. Moreover, the CAT activity showed a negative relationship with K^+^:Na^+^ (*p* < 0.05), Ca^2+^:Na^+^ (*p* < 0.01), and Ca^2+^:Mg^2+^ (*p* < 0.05) in plants.

As shown in **Figure 6B**, the POD, APX, and CAT activities of the leaves were significantly positively related with Na^+^ content (*p* < 0.01), K^+^ content (*p* < 0.01), Ca^2+^ content (*p* < 0.01), and Mg^2+^ content (*p* < 0.01) in the roots during different salt concentration treatments. The relationship between the SOD activity and Ca^2+^ content was also positive (*p* < 0.05). Both the POD activity and the APX activity of the leaves showed a significant negative relationship with K^+^:Na^+^ (*p* < 0.05 and *p* < 0.01), Ca^2+^:Na^+^ (*p* < 0.01 and *p* < 0.01), and Ca^2+^:Mg^2+^ (*p* < 0.05 and *p* < 0.01) in the roots during different salt concentration treatments. Moreover, the CAT activity showed a negative relationship with K^+^:Na^+^ (*p* < 0.05) and Ca^2+^:Na^+^ (*p* < 0.01) in plants.

As shown in **Figure 6C**, the POD and APX activities of the leaves were significantly positively related with Na^+^ content (*p* < 0.01), K^+^ content (*p* < 0.05), Ca^2+^ content (*p* < 0.01), and Mg^2+^ content (*p* < 0.05) in the stems during different salt concentration treatments. The CAT activity of the leaves showed a significant positive relationship with Na^+^ content (*p* < 0.01), K^+^ content (*p* < 0.05), Ca^2+^ content (*p* < 0.01), and Mg^2+^ content (*p* < 0.01) in the stems during different salt concentration treatments. The SOD activity of the leaves showed a significant positive relationship with Ca^2+^ content (*p* < 0.05) in the stems during different salt concentration treatments. Both the POD activity and the APX activity of the leaves showed a significant negative relationship with K^+^:Na^+^ (*p* < 0.01 and *p* < 0.01) and Ca^2+^:Na^+^ (*p* < 0.01 and *p* < 0.01) in the stems during different salt concentration treatments. Similar negative relationships were observed in the CAT activity with K^+^:Na^+^ (*p* < 0.01) and Ca^2+^:Na^+^ (*p* < 0.01). Moreover, the SOD activity showed a negative relationship with Ca^2+^:Mg^2+^ (*p* < 0.01) in the stems.

As shown in **Figure 6D**, the POD and CAT activities of the leaves were significantly positively related with Na^+^ content (*p* < 0.05), K^+^ content (*p* < 0.01), Ca^2+^ content (*p* < 0.01), and Mg^2+^content (*p* < 0.01) in the leaves during different salt concentration treatments. The APX activity of the leaves showed a significant positive relationship with Na^+^ content (*p* < 0.01), K^+^ content (*p* < 0.01), Ca^2+^ content (*p* < 0.01), and Mg^2+^ content (*p* < 0.01) in the leaves during different salt concentration treatments. The SOD activity of the leaves showed a significant positive relationship with K^+^ content (*p* < 0.05) in the stems during different salt concentration treatments. The POD and APX activities of the leaves showed a significant negative relationship with K^+^:Na^+^ (*p* < 0.01 and *p* < 0.01) and Ca^2+^:Na^+^ (*p* < 0.01 and *p* < 0.01) in the leaves during different salt concentration treatments. Similar negative relationships were observed in the CAT activity with K^+^:Na^+^ (*p* < 0.05) and Ca^2+^:Na^+^ (*p* < 0.01).

### Analysis of Antioxidant Enzyme Activities in the Leaves of the Mycorrhizal and Non-mycorrhizal Seedlings During Salt Stress

**FIGURE 7:**
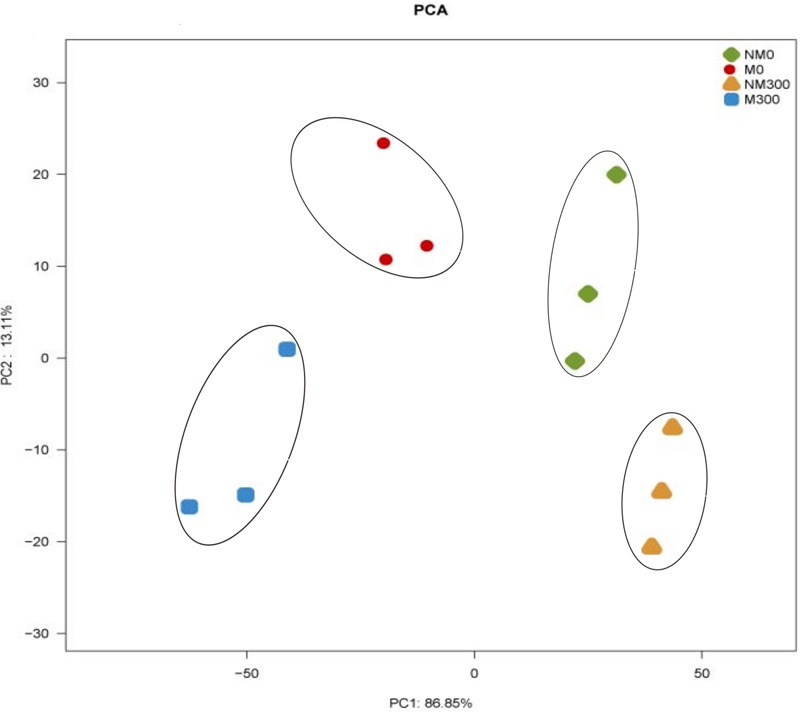
Principal component analysis (PCA) of antioxidant enzyme activities in the leaves of the mycorrhizal (M) and non-mycorrhizal (NM) seedlings during salt stress conditions. 0 mmol/L, without salt stress; 300 mmol/L, during salt stress.

As shown in **Figure 7**, the *X*- and *Y*-axes represent the first (PC1) and second (PC2) principle components, respectively. PC1 and PC2 explained 86.85 and 13.11% of the total variance, respectively. It can be seen that NM0 (antioxidant enzyme activities in the leaves of the non-mycorrhizal seedlings without salt stress), M0 (antioxidant enzyme activities in the leaves of the mycorrhizal seedlings without salt stress), NM300 (antioxidant enzyme activities in the leaves of the non-mycorrhizal seedlings during salt stress) and M300 samples (antioxidant enzyme activities in the leaves of the mycorrhizal seedlings during salt stress) were separated on the basis of the PC1. The cumulative proportion of PC1 was 86.85%. NM0, M0, NM300, and M300 samples were separated on the basis of the PC2. The cumulative proportion of the PC2 was 13.11%. The two PCA axes explained 99.96% of the variation between the different samples. The PCA score plot revealed that the antioxidant enzymic activities in the plants with and without mycorrhizae were different.

## Discussion

### AM Colonization Improved the Seedling Weight During Stress Conditions

The root, stem, and leaf dry weights of the seedlings were significantly inhibited when the plants were subjected to salt stress. However, AM colonization improved the seedling weight during stress conditions. In this study, *E. angustifolia* seedlings inoculated with *R. irregularis* had higher dry weights than the non-mycorrhizal seedlings during salt stress conditions, indicating that the AM symbiosis enhanced salt tolerance. These results were consistent with those from studies on maize (Feng et al., 2002), fenugreek (Evelin and Kapoor, 2014), and Pigeon pea (Garg and Manchanda, 2009). The mycorrhizal seedlings all had higher weights than the non-mycorrhizal seedlings in their studies. Enhanced growth of AM plants has been partly attributed to the ability of the AM fungus to enhance nutrient uptake (Plenchette and Duponnois, 2005; Sharifi et al., 2007; Evelin et al., 2009).

### Inoculation With AMF Increased the SOD, CAT, and APX Activities of Salt-Stressed Plants

Abuscular mycorrhizal symbiosis can increase the activity of antioxidant enzymes, which helps the plants to scavenge ROS generated due to salinity (Garg and Manchanda, 2009; Estrada et al., 2013a). Previous studies have suggested that AMF might enhance antioxidant production in plants and thus protect the plant from oxidative damage during salinity stress conditions (He et al., 2007; Garg and Manchanda, 2009; Hajiboland et al., 2009; Huang et al., 2010; Abdel Latef and Chaoxing, 2011; Borde et al., 2011; Talaat and Shawky, 2011; Li et al., 2012). SOD plays a role in maintaining membrane stability in plant cells and catalyzes the conversion of free O_2_^-^ to O_2_ and H_2_O_2_ (Huang et al., 2010; Estrada et al., 2013a). Our results showed that AM symbiosis significantly influenced the SOD activity in leaves during saline conditions. In the leaf tissues, AM-inoculated seedlings showed significantly higher SOD activity at 300 mmol/L NaCl. Hajiboland et al. (2009) also reported that inoculation with *R. irregularis* increased the SOD activity of salt-stressed tomato plants. Similar results were also found in bajra (Borde et al., 2011) and sustain (Abd Allah et al., 2015).

Because the SOD converts the free O_2_^-^ to H_2_O_2_, it is necessary to scavenge H_2_O_2_. Other important antioxidant enzymes such as CAT, APX, and POD were also examined in this study. CAT, APX, and POD are essential for plants to tolerate salinity because these enzymes decompose H_2_O_2_ to oxygen and water. In this study, the effect of AMF on CAT activity during salinity stress was significant, which is consistent with other studies indicating that AM symbiosis enhanced the CAT activity of salt stressed seedlings of plants such as pigeon pea, tomato, and suaeda (Garg and Manchanda, 2009; Huang et al., 2010; Li et al., 2012).

The greater APX activity that we observed in mycorrhizal seedlings suggests that APX could be related to the salt tolerance of the plant, and the enhanced APX activity may increase the ability of the host plant to scavenge H_2_O_2_ and enhance mycorrhizal seedling growth during salinity. The results are similar in part to those obtained by Hajiboland et al. (2009) and Abdel Latef and Chaoxing (2011) who reported greater APX activity of salt-stressed tomato inoculated with *G. intraradices* or *G. mosseae.*

This study revealed that the activities of SOD, CAT, and APX within the plants were significantly higher in the AMF than the non-AMF plants during salt stress conditions with the exception of POD activity. These results illustrate that AM symbiosis can help the plants protect themselves from the oxidative effects of the ROS. SOD, CAT, and APX are metalloenzymes whose activities depend on the availability of micronutrients (Evelin et al., 2009). The higher SOD, CAT, and APX activity in mycorrhizal plants may be associated with the enhanced plant growth and acquisition of nutrients (Alguacil et al., 2003). In addition, the two-way ANOVA data in this study revealed that AMF does not have a significant effect on the POD activity in the *E. angustifolia* seedlings during salt stress. Therefore, our results were not completely consistent with previous reports where AM symbiosis affected the POD activity of plant to withstand oxidative stress (He et al., 2007; Borde et al., 2011). The main reason for this finding is that the detoxification of ROS also involves other antioxidant enzymes such as GR, and antioxidants such as ascorbate, glutathione, and tocopherols merit further studies using mycorrhizal plants during salt stress conditions. On the other hand, the fact that different AM fungal species colonize different host plants can also lead to different antioxidant enzyme activities during salt stress conditions (Evelin et al., 2009).

### AM Colonization Increased K^+^, Ca^2+^, and Mg^2+^ Accumulation and Mitigated NaCl-Induced Ionic Imbalances in Salt-Stressed Plants

In this study, salinity stress increased the Na^+^contents of roots and leaves in non-mycorrhizal seedlings, because plants tend to take up more Na^+^ when salt concentrations in the soil are high (Evelin et al., 2009). Salinity stress increased the K^+^, Ca^2+^, and Mg^2+^ contents of the roots and leaves in non-mycorrhizal seedlings partly due to the mechanism of abiotic stress tolerance of *E. angustifolia*. Inoculation with AM fungus promotes Na^+^, K^+^, Ca^2+^, and Mg^2+^ accumulation in the roots, stems, and leaves compared to those of the non-inoculated seedlings during salt and no salt stress treatments. The reason for this could be that AM symbioses often have not only increased length but also modified root architecture via the extraradical mycelia that increase mineral nutrient elements uptake. Also, AM fungus can selectively take up elements such as K^+^, Ca^2+^, and Mg^2+^, all of which act as osmotic equivalents while avoiding uptake of Na^+^ (Hammer et al., 2011).

Our study also showed that the Na^+^(stem + leaf) to Na^+^(root) ratio was significantly lower in mycorrhizal seedlings than in non-mycorrhizal seedlings during salt stress, suggesting that the translocation of Na^+^ from the roots to the aerial parts was restricted in mycorrhizal seedlings as a strategy to limit the accumulation of this toxic ion in photosynthetic tissues (Porcel et al., 2016). Some previous studies also observed that there was an obvious increase in the Na^+^ shoot to root ratio due to the salinity in non-mycorrhizal seedlings, while the increase in Na^+^ shoot to root ratio was lower in mycorrhizal seedlings (Evelin et al., 2012; Porcel et al., 2016).

Na^+^ directly competes with K^+^ for binding sites that are essential for various metabolic functions (Evelin et al., 2009), thus maintaining a high cytosolic K^+^:Na^+^ ratio is a feature of salt tolerance in plants. According to some researchers, AM symbiosis prevents excess uptake of Na^+^ by the root tissues, but enhancing K^+^ absorption during saline conditions may help to maintain a high K^+^:Na^+^ ratio (Evelin et al., 2013). Our study showed that inoculation with AMF could enhance Na^+^ accumulation in roots, stems, and leaves, which is consistent with reports that AMF sometimes enhances plant Na^+^ uptake (Allen and Cunningham, 1983; Mardukhi et al., 2011). The increased K^+^ accumulation in the roots during salinity stress conditions may result in the transfer of higher amounts of K^+^ to the stems and leaves. Our study suggested that the mycorrhizal plants significantly promoted K^+^ accumulation in the roots, stems, and leaves during salt stress conditions, resulting in increased K^+^:Na^+^ ratios in the leaves of mycorrhizal *E. angustifolia* seedlings.

Salt stress has been reported to inhibit Ca^2+^ uptake and transport in roots, inducing a lower Ca^2+^:Na^+^ ratio (Grattan and Grieve, 1998; Evelin et al., 2013; Hajiboland, 2013). Ca^2+^ participates in important processes that preserve the structural and functional integrity of plant membranes, stabilize cell wall structures, and regulate ion transport and selectivity (Maathuis and Amtmann, 1999). This study showed that AMF inoculation significantly promoted the accumulation of Ca^2+^ in root and leaf tissues at 300 mmol/L NaCl. Evelin et al. (2013) reported that higher Ca^2+^ uptake in mycorrhizal plants may mitigate NaCl-induced ionic imbalances.

The major function of Mg^2+^ in green leaves is to serve as the central atom of the chlorophyll molecule. A reduction in Mg^2+^ uptake may reduce chlorophyll concentrations and photosynthesis in leaves, resulting in a reduction in plant growth (Giri and Mukerji, 2004). In this study, AM colonization showed a positive relationship with Mg^2+^ concentrations in the roots, stems, and leaves. Mycorrhizal symbioses reduce the negative effect of Na^+^ by facilitating the absorption of Mg^2+^. The uptake of Mg^2+^ can be strongly depressed by Ca^2+^. It has been reported that the mycorrhizal plants maintain higher Ca^2+^:Mg^2+^ ratios than non-mycorrhizal plants (Evelin et al., 2013). However, in this study, we found that the Ca^2+^:Mg^2+^ ratios in the stem and leaf tissues of mycorrhizal plants decreased in response to salinity, and those in the roots had no obvious differences between the mycorrhizal and non-mycorrhizal plants during NaCl stress. Inoculation with AM fungi alleviated the competition between Mg^2+^ and Ca^2+^. This may be attributed to a more efficient uptake of Mg^2+^ and the mitigation of the Mg^2+^ and Ca^2+^ ionic imbalance.

### Correlation of Antioxidant Enzyme Activities and Ion Distribution During Salt Treatments

In this study, the significant positive correlation between antioxidant enzyme activities (SOD, POD, APX, and CAT) and ion (K^+^, Ca^2+^, and Mg^2+^) content indicated that AM symbiosis alleviates the detrimental effects of salinity on plant growth by improving plant mineral nutrition and increasing the activity of antioxidant enzymes (**Figures 6A–D**). The reason is that AM symbiosis increased ion (K^+^, Ca^2+^, and Mg^2+^) uptake and improved the integrity and stability of cellular membranes that facilitate the maintenance of a higher electrolyte concentration in plants grown during saline conditions (Chandrasekaran et al., 2014). Alternatively, AM symbiosis and salt stress increased the SOD, CAT, and APX activities in mycorrhizal plants, which were consistent with previous reports on the meta-analysis of AM effects on plants grown during salt stress conditions (Chandrasekaran et al., 2014).

### Effect of AM Symbiosis on Antioxidant Enzyme Activities in the Leaves During Salt Stress Conditions

In this study, the results of the PCA showed that the AM symbiosis significantly affect the SOD, CAT, POD, and APX activities in plants (**Figure 7**). There have been several studies supporting the view that enzymatic activities are significantly increased in mycorrhizal compared to non-mycorrhizal plants during salt stress conditions (Alguacil et al., 2003; Ghorbanli et al., 2004).

## Conclusion

In summary, our findings in this study show that the biomass of the *E. angustifolia* seedlings could be increased by inoculation with *R. irregularis*. AMF can help protect *E. angustifolia* seedlings against salt stress conditions. *R. irregularis* notably improved the capacity of *E. angustifolia* seedlings to mitigate oxidative stress during excessive soil salinity. The inoculation of AMF alleviates the deleterious effects of salt stress by increasing K^+^, Ca^2+^, and Mg^2+^ accumulation and maintaining higher K^+^:Na^+^ in leaves and lower Ca^2+^:Mg^2+^ ratios in plants compared to the non-mycorrhizal seedlings. *R. irregularis* has great potential to enhance the biomass of *E. angustifolia* seedlings in saline soils. In addition, the data from this study support further studies on the responses of the AM symbiosis to salinity.

## Author Contributions

Experiments were designed by WC and F-QS. Statistical analysis was performed by WC, XS, and T-TJ. The manuscript was written by WC. The manuscript was revised by XS, X-XF, and FS. All authors read and approved the final manuscript.

## Conflict of Interest Statement

The authors declare that the research was conducted in the absence of any commercial or financial relationships that could be construed as a potential conflict of interest.
